# From Biomarkers to Biosensors: Transforming Comorbidity Management in Dialysis Care

**DOI:** 10.3390/s26061929

**Published:** 2026-03-19

**Authors:** Ali Fardoost, Koosha Karimi, Aratrika Bhattacharya, Viresh Patel, Matthew Lucien Saintyl, Samanthia Grace Welsh, Mehdi Javanmard

**Affiliations:** Department of Electrical and Computer Engineering, Rutgers University, Piscataway, NJ 08854, USA; af1069@scarletmail.rutgers.edu (A.F.); kk1301@scarletmail.rutgers.edu (K.K.); ab3084@scarletmail.rutgers.edu (A.B.); vdp45@scarletmail.rutgers.edu (V.P.); mls463@scarletmail.rutgers.edu (M.L.S.); sgw52@scarletmail.rutgers.edu (S.G.W.)

**Keywords:** dialysis, chronic kidney disease (CKD), comorbidities, parathyroid hormone (PTH), *β*_2_-microglobulin, creatinine, cystatin C, biosensors

## Abstract

Patients receiving dialysis treatments suffer from a high rate of systemic comorbid conditions, including cardiovascular disease, mineral and bone disorders, chronic inflammation, amyloidosis, and recurring infections, leading to increased morbidity and mortality rates despite the progress made in the field of renal replacement therapies. The aforementioned conditions result from the continued dysregulation and overproduction of molecular biomarkers, which cannot be adequately monitored by traditional, intermittent laboratory tests. This review critically assesses the newly developed biosensor technologies for the detection of major dialysis biomarkers, including potassium, phosphorus, parathyroid hormone (PTH), *β*_2_-microglobulin, creatinine, and cystatin C, with special emphasis on biosensors based on electrochemistry, optics, impedimetry, nanophotonics, and biological engineering techniques. These recent biosensors have been evaluated based on their analytical performance, the biofluids used in the studies, and their suitability for measuring relevant concentrations of these biomarkers. Special attention is given to biosensors capable of continuous operation or minimally invasive sampling, as well as to newly developed biofluid sampling techniques, including microneedle-, microtube-, and micropillar-based systems, for the long-term monitoring of the biomarkers in the serum of patients receiving dialysis treatments. The biosensing techniques for measuring infection biomarkers have also been discussed, given the high risk of bloodstream and access infections among patients receiving dialysis. The limitations of these biosensors include biofouling, calibration drift, and their integration into the dialysis treatment workflow. Finally, the future prospects of the recent biosensors offer the possibility of the proactive management of the high rate of comorbid conditions in this high-risk population of patients receiving dialysis treatments.

## 1. Introduction

Dialysis is a life-sustaining therapy for patients with advanced renal dysfunction, serving as an artificial replacement for kidney function when the kidneys are no longer able to effectively remove metabolic waste products, excess fluids, and electrolytes from the bloodstream. It is most commonly employed in end-stage renal disease (ESRD), typically when kidney function declines below approximately 15% [1], but is also used in cases of acute kidney injury (AKI) resulting from trauma, infection, ischemia, or nephrotoxic exposure [2,3]. While dialysis has significantly improved survival among patients with renal failure, it does not fully replicate the regulatory, endocrine, and immunological functions of healthy kidneys. As a result, patients undergoing dialysis remain highly vulnerable to a wide range of systemic complications that contribute to elevated morbidity and mortality.

Two primary dialysis modalities are currently used in clinical practice: peritoneal dialysis (PD) and hemodialysis (HD). Peritoneal dialysis utilizes the peritoneal membrane as a semipermeable filter, where a dialysate solution is introduced into the abdominal cavity via a catheter and allowed to dwell while waste products diffuse from the blood into the dialysate. The dialysate is then drained and replaced, either manually through continuous ambulatory peritoneal dialysis (CAPD) or automatically using a cycler in automated peritoneal dialysis (APD) [4,5]. In contrast, hemodialysis involves extracorporeal circulation of blood through a dialyzer, where solute exchange and ultrafiltration occur across a synthetic membrane before the cleansed blood is returned to the patient. Hemodialysis is typically performed two to three times per week in specialized centers, though home-based modalities are increasingly adopted [6,7].

Both PD and HD are renal replacement therapies primarily indicated for patients with end-stage renal disease (ESRD), a condition characterized by irreversible and severe decline in kidney function, typically defined by a glomerular filtration rate (GFR) of less than 15 mL/min/1.73 m^2^ [8]. In ESRD, the kidneys are no longer able to adequately remove metabolic waste products, regulate electrolyte balance, or maintain fluid homeostasis [3]. Peritoneal dialysis serves as an alternative pathway to partially compensate for the lost kidney function by facilitating solute clearance and fluid removal through the peritoneal membrane, thereby helping to maintain metabolic stability and improve patient survival and quality of life [9,10].

Despite their effectiveness in removing urea and excess fluid, both dialysis modalities are associated with substantial physiological stress and long-term complications. Dialysis patients face a disproportionately high risk of cardiovascular disease, bone and mineral disorders, chronic inflammation, amyloidosis, and recurrent infections, which collectively account for the majority of dialysis-related hospitalizations and deaths [11,12,13,14]. These comorbidities arise from persistent biochemical imbalances, incomplete toxin clearance, and disrupted endocrine signaling that cannot be fully corrected by conventional dialysis protocols. Importantly, many of these complications develop insidiously, often progressing undetected between dialysis sessions until they reach clinically severe or life-threatening stages.

Central to the development and progression of dialysis-related comorbidities is the accumulation and dysregulation of specific molecular biomarkers [15]. Electrolytes such as potassium and phosphorus, hormones such as parathyroid hormone (PTH), middle-molecular-weight proteins like *β*_2_-microglobulin, and traditional markers of renal function, including creatinine and cystatin C, serve as critical indicators of dialysis adequacy and systemic homeostasis [16,17,18]. Abnormal levels of these biomarkers are directly linked to adverse outcomes, including fatal cardiac arrhythmias, vascular calcification, renal osteodystrophy, dialysis-related amyloidosis, and increased cardiovascular mortality [19,20]. However, many of these biomarkers exhibit rapid temporal fluctuations during and between dialysis sessions, particularly during the interdialytic interval, when patients are at greatest risk [21].

The goal of this study is to highlight biomarkers indicative of interrelated biochemical pathways that overall contribute to the common comorbidities of dialysis patients. Potassium, phosphorus, PTH, *β*_2_-microglobulin, creatinine, and cystatin C do not stand alone as indicators; rather, they form an interconnected network that describes electrolyte homeostasis, mineral metabolism, uremic toxin accumulation, and dialysis adequacy [16,18]. The dysregulation of each of these pathways contributes directly to the development of cardiovascular disease, bone disease, and amyloidosis, as well as enhancing the risk of systemic inflammation and mortality [19]. Classical inflammatory markers, such as interleukin-6 (IL-6) and C-reactive protein, can be useful clinically; however, they are more so downstream markers of inflammation than direct dialysis modifiers via clearance capabilities [20]. Therefore, the purpose here is to identify and provide a rationale for the attention of biomarkers/measures that have mechanistic interconnections and actionable opportunities via dialysis-based interventions, making them optimal candidates for the development of novel biosensing technologies (both continuous and integrated).

Conventional monitoring of dialysis patients relies primarily on intermittent blood sampling and centralized laboratory analysis, which inherently limits temporal resolution and delays clinical intervention. As a result, dangerous biomarker excursions may go undetected until symptoms manifest or irreversible damage has occurred. This reactive approach contrasts sharply with the growing clinical need for continuous, real-time physiological insight that can support proactive and personalized dialysis management. Continuous biomarker monitoring has the potential to inform dynamic adjustments in dialysis duration, frequency, dialysate composition, and pharmacological intervention, thereby improving treatment efficacy and reducing complication rates [13,14].

Though potassium, phosphorus, parathyroid hormone (PTH), *β*_2_-microglobulin, creatinine, and cystatin C are commonly used as independent biomarkers in clinical practice, the dysregulation of these biomarkers in CKD patients undergoing dialysis appears to follow similar pathophysiological pathways [22]. Reduced GFR in CKD patients increases the levels of creatinine, cystatin C, and *β*_2_-microglobulin in a similar manner. Numerous studies have confirmed a strong intercorrelation among these filtration markers. The combined levels of these markers are useful in evaluating the severity of CKD [23]. Phosphorus retention in CKD patients is a major cause of secondary hyperparathyroidism through the PTH–phosphorus axis. This axis is related to reduced kidney function. Electrolytes such as potassium are commonly found in reduced amounts in CKD patients. Hyperkalemia in CKD patients is a cause of cardiovascular instability [24,25]. All these biomarkers are related to a similar mechanism in CKD patients.

Recent advances in biosensor technology have opened new pathways for addressing these limitations. Innovations in electrochemical, optical, impedimetric, and nanomaterial-enhanced biosensing platforms have enabled sensitive, rapid, and increasingly miniaturized detection of clinically relevant biomarkers from blood, dialysate, and alternative biofluids [26,27,28,29]. Many of these systems are compatible with point-of-care testing, wearable formats, or direct integration with dialysis hardware, enabling continuous or near-continuous monitoring without disrupting existing treatment workflows. Importantly, biosensors align with the broader shift toward precision medicine, enabling patient-specific monitoring strategies that reflect individual physiology rather than population-averaged thresholds [30,31,32].

Significant advancements in wearable biosensors have been strongly correlated with the development of minimally invasive biofluid sampling techniques that facilitate continuous or near-continuous biofluid monitoring. Among these techniques, microneedle, microtube, and micropillar-based integrated systems have been recognized as promising approaches for long-term biofluid monitoring while maintaining access to interstitial fluid (ISF), enabling dynamic biomarker monitoring in the bloodstream without the need for routine blood sampling [26,33]. Specifically, microneedle-based systems can be employed for the painless sampling of ISF through the stratum corneum layer while integrating with electrochemical/optical biosensors for long-term monitoring with high patient compliance. The microtube and micropillar-based architectures can be employed as alternative techniques for improving fluidic transport while increasing the stability for long-term sampling [33]. When combined with miniaturized sensing components and wireless sensing electronics, these techniques can be recognized as a promising route for the development of continuous biofluid monitoring in real-world environments. These sampling–sensing-based techniques can be recognized as essential platforms for the development of wearable biosensors, including dialysis care and long-term monitoring for chronic diseases [33,34].

In this review, we examine the role of biosensors and biomarkers in managing dialysis-related comorbidities, with a focus on potassium, phosphorus, parathyroid hormone, *β*_2_-microglobulin, creatinine, and cystatin C due to their strong clinical relevance. We discuss current biosensing strategies employed for their detection, including electrochemical, optical, nanophotonic, and biologically engineered platforms, and evaluate their potential for real-time, minimally invasive monitoring. In addition, we address emerging biosensors for bacterial and infection-related biomarkers, which are critical given the heightened susceptibility of dialysis patients to bloodstream infections. Finally, we analyze the limitations of existing technologies and highlight future directions aimed at integrating biosensors into next-generation dialysis systems to enable safer, more responsive, and more personalized patient care.

Several review articles have focused on biomarkers or biosensor technologies for chronic kidney disease (CKD) and dialysis. For example, Tricoli and Neri reviewed the miniaturized sensor technology for the monitoring of CKD patients, with a particular emphasis on conventional biomarkers such as creatinine and urea [35]. Recently, other review articles have focused on biosensors for the diagnosis of CKD [36], electrochemical biosensors for the diagnosis of CKD [37], and advances in electrochemical biosensors for the detection of kidney biomarkers [38]. However, these review articles primarily focus on the static detection technologies or the conventional sensing modalities. In contrast, this review offers a comprehensive and mechanistic overview of key biomarkers associated with the dialysis process, including electrolytes, middle-molecular-weight toxins, hormonal regulators, and infection biomarkers, along with emerging biosensors for real-time, minimally invasive monitoring of these biomarkers.

## 2. Biomarkers and Biosensors

In dialysis patients, routine blood work is essential for monitoring toxins and metabolic byproducts that the kidneys filter normally. These biomarkers, potassium, phosphorus, parathyroid hormone, *β*_2_-microglobulin, cystatin C, and creatinine, among many others, serve as critical indicators of dialysis efficacy and overall patient health. When these compounds accumulate due to impaired renal clearance, they contribute to a cascade of life-threatening complications, making their regular assessment indispensable in managing end-stage renal disease.

In order to detect these types of biomarkers, conventional gold-standard methods are commonly employed. For molecular biomarkers such as cytokines and protein indicators, enzyme-linked immunosorbent assay (ELISA) remains the clinical gold standard due to its high specificity and quantitative capability; however, ELISA typically requires centralized laboratory infrastructure, trained personnel, multiple incubation and washing steps, and several hours to generate results [39,40]. Similarly, bacterial biomarkers are traditionally identified using microbiological culture techniques, which, although highly reliable and capable of confirming viable organisms, often require 24–72 h for colony growth and subsequent analysis, thereby delaying clinical decision-making [41,42].

In contrast, researchers have developed biosensors to enable simple, efficient, and rapid analyses at low cost. Patients would only need to provide a small sample of blood, saliva, or urine and could obtain results within minutes rather than hours or days. These biosensors are particularly advantageous in regions with limited laboratory infrastructure and in clinical settings where rapid decision-making is critical for optimizing dialysis treatment. With faster and potentially point-of-care monitoring technologies, medical professionals can more promptly assess biomarker fluctuations and adjust dialysis regimens to improve patient outcomes.

The choice of biofluid significantly influences the analytical performance, clinical applicability, and patient acceptability of biosensing platforms in dialysis care. Blood is the current clinical reference standard biofluid of choice due to its high analyte concentration and well-established baseline levels of biomarkers. Blood biofluids provide a direct measure of physiological status. However, blood biofluid sampling is invasive. Saliva and urine biofluids have been studied and shown to have potential for monitoring renal function markers. For example, correlations between salivary and serum creatinine and urea levels have been found in patients with chronic kidney disease. However, there are many inconsistencies and limitations in analytical sensitivity [43]. Interstitial fluid (ISF) biofluid has been found to have great potential as a biofluid for wearable sensors. This is due to the similarity in composition between ISF and blood biofluids [44]. ISF biofluid can be obtained minimally invasively using microneedles [45]. This provides a valuable opportunity to continuously monitor homeostatic markers. However, ISF biofluid has yet to be implemented in clinical settings. Biofluid-specific problems and their solutions are yet to be addressed. Urine biofluid has many limitations and is not suitable for monitoring homeostatic markers. This is especially true for anuric and oliguric dialysis patients. No biofluid has been found to have universal potential. Each biofluid has been found to have advantages and limitations. Biofluid selection is dependent on the intended use.

### 2.1. Molecular Biomarkers

#### 2.1.1. Potassium

Potassium, a key electrolyte, must be tightly regulated because even slight elevations (hyperkalemia) can destabilize cardiac cell membranes, leading to fatal arrhythmias such as ventricular tachycardia or fibrillation. In ESRD, impaired renal potassium excretion leads to progressive potassium accumulation, which is further modulated by intermittent dialysis therapy. Dialysis patients are particularly vulnerable during the interdialytic period when potassium intake (from diet or tissue breakdown) outpaces removal. Without adequate dialysis, potassium levels rise insidiously, often without symptoms, until sudden cardiac arrest occurs. Conversely, hypokalemia (low potassium), though less common, can result from overzealous dialysis or malnutrition, causing muscle weakness and further arrhythmia risks. Thus, potassium monitoring is a delicate balancing act, requiring frequent checks to prevent catastrophic cardiac events [46,47,48].

Traditional monitoring of Potassium relies on sporadic blood tests, leaving dangerous fluctuations undetected [49]. To address this, three complementary biosensing technologies have emerged, each targeting different aspects of dialysis care: continuous interdialytic monitoring, real-time intradialytic tracking, and low-cost disposable sensing.

**Figure 1:**
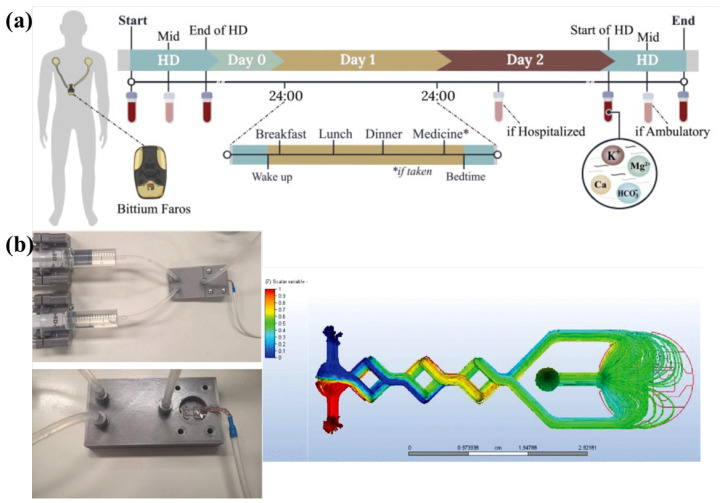
(**a**) The data acquisition protocol used in wrist-worn biosensor for potassium monitoring presented by Rodrigues et al. [46]. (**b**) The flow simulation and chamber connected to syringe pumps with cover (**in middle**), and without cover, to show the sensor placement (**bottom**) that is presented by Wildner et al. [47].

The first approach, presented by Rodrigues et al. a wrist-worn biosensor combining ECG and bioimpedance measurements, offers noninvasive, continuous potassium estimation outside clinical settings [46]. This device leverages two physiological signals: (1) ECG waveform alterations (since potassium directly affects cardiac repolarization, seen in peaked T-waves during hyperkalemia) and (2) bioimpedance shifts (changes in tissue electrical resistance correlated with potassium concentration). By cross-validating these signals, the system achieves blood-test-level accuracy over 72 h, providing early warnings before symptoms manifest [46]. This is particularly valuable for the interdialytic gap, where most hyperkalemia-related deaths occur. However, while this method excels in ambulatory monitoring, dialysis sessions require higher temporal resolution and direct blood-contact sensing to guide real-time treatment adjustment. The data acquisition protocol of this biosensor is presented in Figure 1a.

This leads to the second innovation: an implantable iridium oxide microprobe with anti-fouling properties (Iridium Oxide-Based Potassium Sensitive Microprobe) conducted by Wldner et al. Unlike conventional sensors that degrade due to protein fouling, this probe uses a zwitterionic polymer coating, a material with balanced positive and negative charges that repels proteins, enabling stable potentiometric detection for over 28 days. The sensor operates by measuring voltage changes as potassium ions interact with the iridium oxide electrode, providing continuous intravascular readings when integrated into dialysis catheters [47]. This is transformative for intradialytic care, where real-time potassium data could allow dynamic dialysate adjustments, preventing rebound hyperkalemia post-treatment. However, implantable probes, while precise, may not be practical for all patients due to cost and invasiveness, necessitating a third, disposable alternative for widespread use. The flow simulation in the biosensor and the 3D view of chamber with and without cover is illustrated in Figure 1b.

In another study, Depauw et al. developed a highly selective fluorescent potassium (K^+^) sensor designed for real-time detection of potassium in living tissues, addressing the challenge of distinguishing K^+^ from competing physiological ions such as Na^+^, which is present at significantly higher concentrations in biological environments [50]. The sensor is based on a supramolecular design incorporating a triazacryptand-based ionophore with strong and selective binding affinity for potassium, coupled to a fluorophore that enables optical signal transduction upon ion binding. The sensing mechanism relies on potassium-induced modulation of the photophysical properties of the fluorophore, resulting in a measurable fluorescence response proportional to K^+^ concentration. Notably, the probe demonstrated high selectivity over sodium and other biologically relevant cations, rapid response kinetics, and compatibility with physiological conditions. The authors validated the sensor in live-cell and tissue imaging experiments, demonstrating its ability to monitor dynamic potassium fluxes in complex biological systems. This work represents a significant advancement in potassium biosensing by combining molecular selectivity, real-time imaging capability, and applicability in living tissues, thereby offering potential utility for biomedical applications requiring precise electrolyte monitoring.

Together, these technologies form a multi-modal potassium management framework. Future integration with pH-sensitive ISFETs (e.g., from Drain Current Centric Modality) could further enhance safety by detecting concurrent acidosis, a common contributor to dialysis-related arrhythmias. By combining these approaches, clinicians could move from reactive to predictive care, personalizing dialysis prescriptions and dietary guidance based on continuous, context-rich electrolyte data, ultimately reducing cardiac mortality in this high-risk population.

#### 2.1.2. Phosphorus

Phosphorus homeostasis is another primary concern in ESRD, as failing kidneys cannot excrete excess dietary phosphate. Elevated phosphorus (hyperphosphatemia) binds with calcium to form insoluble deposits in soft tissues, leading to vascular calcification, a key driver of cardiovascular mortality in dialysis patients. Additionally, high phosphorus triggers secondary hyperparathyroidism by stimulating excessive parathyroid hormone (PTH) release, which we will discuss next. To mitigate these effects, dialysis must efficiently remove phosphate, and patients often require phosphate-binding medications to limit gastrointestinal absorption. However, even with treatment, phosphorus remains a stubborn biomarker to control, and its accumulation accelerates atherosclerosis and bone demineralization, contributing to fractures and cardiovascular events [51,52,53].

Phosphate monitoring plays a vital role in clinical management, particularly for patients with chronic kidney disease (CKD) and those undergoing dialysis, where abnormal phosphate levels can lead to severe cardiovascular and metabolic complications [54,55]. Conventional laboratory-based assays, though accurate, are time-consuming and unsuitable for continuous or point-of-care monitoring [56,57]. To address this limitation, recent research has focused on developing biosensor platforms capable of rapid, sensitive, and miniaturized phosphate detection compatible with biological samples. In 2024–2025, several innovative approaches have emerged ranging from enzyme-based electrochemical biosensors and ion-selective electrodes to genetically engineered whole-cell biosensors that demonstrate promising sensitivity, portability, and adaptability for real-time phosphate analysis. The following studies highlight these technological advances and their potential relevance for clinical phosphate monitoring in dialysis care.

**Figure 2:**
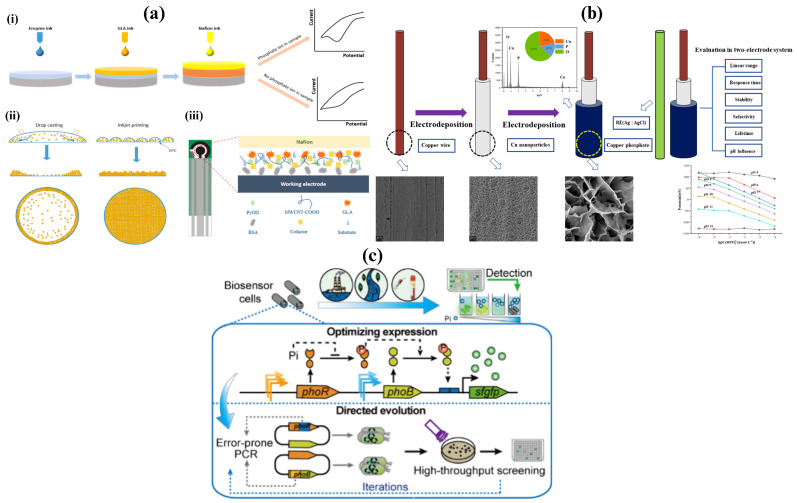
(**a**) Enzyme crosslinking through inkjet printing on reagentless biosensors [58]. (**i**) Illustration of the surface modification steps on the working electrode and the corresponding sensing mechanism of the biosensor. (**ii**) Visualization of droplet drying behavior on the electrode surface, comparing particle distribution patterns obtained via drop casting and inkjet printing methods (side and top views). (**iii**) Cross-sectional schematic representation of the functionalized working electrode structure. (**b**) The schematic diagram of the all-solid-state copper-based ion-selective electrode (ISE) fabrication process and the SEM images of the surface in different fabrication steps [59]. (**c**) The schematic illustrating the mechanism and working principle of the PhoR-PhoB-based biosensor cells for Phosphorus detection [60].

Zhang et al. [58] developed a reagentless enzyme-based electrochemical biosensor for phosphate detection using an inkjet printing technique to immobilize pyruvate oxidase directly on screen-printed electrodes, showed in Figure 2a. This method eliminated the need for manual enzyme coating, improving reproducibility and scalability for point-of-care use. The biosensor operates by measuring the current generated from the enzymatic reaction between phosphate and pyruvate oxidase, which produces an electroactive species proportional to phosphate concentration. The device demonstrated a broad linear range covering physiological serum phosphate levels, high reproducibility (RSD < 4%), and excellent recovery (98.9–103%) in artificial serum using only a 30 μL sample. The printed sensor also retained about 89% of its initial response after three weeks, highlighting good stability and potential applicability for rapid phosphate monitoring in dialysis patients.

Moreover, He et al. [59] designed an all-solid-state copper-based ion-selective electrode (ISE) for rapid phosphate detection (Figure 2b). The innovation lies in its electrochemically formed copper-phosphate nanostructured layer that provides high sensitivity and stability without requiring an internal solution. The biosensor functions by detecting potential changes at the copper-phosphate interface as phosphate ions interact with the surface, following Nernstian behavior. The electrode exhibited a detection limit of approximately 1 μM, a fast response time (<10 s), and excellent selectivity against common interfering ions. It remained stable for over two months and performed well in real water samples. Although designed for environmental applications, its sensitivity, rapid response, and operation near neutral pH make it a promising platform for future adaptation to serum phosphate sensing in dialysis patients.

Furthermore, recently, Cao et al. [60] developed a genetically engineered whole-cell biosensor based on the PhoR–PhoB two-component regulatory system to detect ultralow concentrations of inorganic phosphate. Their innovation involved using directed evolution to enhance the phosphate sensitivity of the PhoR–PhoB signaling pathway in *E. coli*, enabling detection at levels far below those of natural bacterial regulation. The biosensor works by coupling phosphate-induced activation of the PhoB transcription factor to the expression of a fluorescent reporter gene, where fluorescence intensity directly reflects phosphate concentration. The working principle and a schematic on the biosensor cell are presented in Figure 2c. Through iterative mutagenesis and screening, the authors achieved a detection limit down to the nanomolar range, significantly improving upon existing biological phosphate sensors. The optimized strain exhibited high specificity, rapid response, and stability across various environmental and physiological conditions. While primarily demonstrated in aqueous samples, this engineered PhoR–PhoB biosensor offers a powerful platform for sensitive phosphate monitoring in biological fluids, with strong potential for clinical applications such as tracking phosphate imbalance in dialysis patients.

Together, these three biosensing strategies represent significant progress toward achieving fast, sensitive, and accessible phosphate detection. The inkjet-printed enzyme biosensor introduced by Zhang et al. [58] offers a practical, reagentless, and cost-effective approach suitable for integration into portable diagnostic devices. The copper-based ion-selective electrode developed by He et al. [59] provides excellent stability and rapid response, highlighting the feasibility of long-term, real-time sensing applications. Meanwhile, the PhoR–PhoB whole-cell biosensor by Cao et al. [60] demonstrates the power of synthetic biology to detect ultralow phosphate concentrations with exceptional specificity. Looking forward, future research should aim to adapt these biosensors for direct analysis of human serum and real-time monitoring during dialysis sessions, addressing challenges such as biofouling, calibration drift, and matrix interference. Integration with microfluidic systems and wireless readouts could further enable continuous phosphate monitoring, paving the way toward fully automated and patient-friendly diagnostic tools for improved management of phosphate imbalance in dialysis patients.

#### 2.1.3. Parathyroid Hormone (PTH)

Parathyroid hormone (PTH), produced by the parathyroid glands, becomes dysregulated in ESRD due to phosphorus retention and vitamin D deficiency, both consequences of kidney failure. Chronically elevated PTH (secondary hyperparathyroidism) leads to renal osteodystrophy, a bone disorder characterized by excessive resorption and abnormal mineralization, causing pain, deformities, and fractures. Moreover, high PTH levels exacerbate vascular calcification by promoting calcium release from bones into the bloodstream. While dialysis can partially correct the metabolic disturbances contributing to PTH overproduction, many patients still require calcimimetics (e.g., cinacalcet) or surgical parathyroidectomy in severe cases. Thus, PTH serves as both a marker of bone metabolism and a predictor of cardiovascular risk in dialysis patients [61,62].

The clinical need to manage parathyroid hormone (PTH) levels in dialysis patients, particularly those with chronic kidney disease-mineral and bone disorder (CKD-MBD), has driven the development of a broad range of biosensing technologies [63]. These encompass electrochemical, optical, and hybrid methods, offering new possibilities for real-time, point-of-care (POC), and intraoperative PTH monitoring.

**Figure 3:**
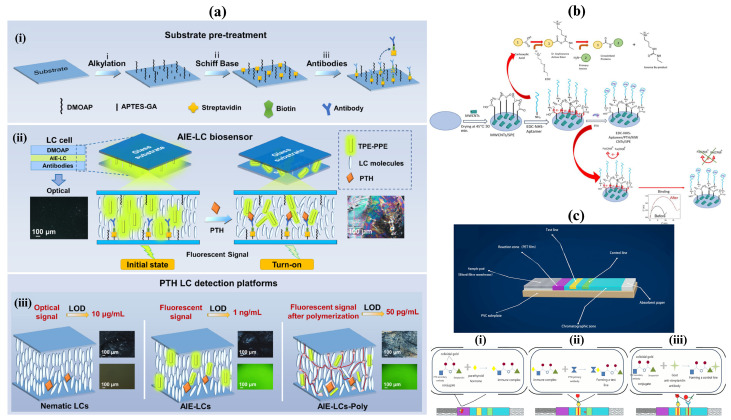
(**a**) Schematic diagram of LC-based biosensor for PTH. (**i**) Construction process of LC biosensor substrate. (**ii**) Schematic of AIE-LCs alignment and the corresponding POM images before the introduction of PTH (**left**), and after the introduction of PTH (**right**). (**iii**) Schematic of LC alignment in different platforms after the introduction of PTH. [64]. (**b**) Schematic view of aptamer-based electrochemical biosensor system [65]. (**c**) Principle and detection procedures of the PTH-ICGT assay [66]. Components of a test strip on top. (**i**) The PTH in the sample binds to the colloidal gold–antibody on the conjugate pad. (**ii**) Through capillary action, colloidal gold-labeled complexes are captured by the solid-phase chromogenic agent (PTH antibody) located in the test zone on the membrane to form a pink or dark red ribbon. (**iii**) Finally, un-reacted complexes stagnate at the control zone to form a control line by binding to pre-coated anti-streptavidin antibody on the membrane.

Tong et al. [64] developed an aggregation-induced emissive liquid crystal–polymer (AIE-LC-Poly) composite membrane for the dual-channel detection of parathyroid hormone (PTH). Figure 3a shows the diagram and working principle of the biosensor. The system integrates AIE-active luminogens within a liquid-crystal polymer matrix, allowing simultaneous fluorescence and optical signal transduction. Upon PTH binding, molecular aggregation within the membrane induces a strong fluorescence enhancement (AIE effect) alongside a measurable change in the liquid crystal orientation, enabling both visual and quantitative readouts. This dual-mode platform provides high sensitivity and reliability for PTH monitoring. The membrane achieved a rapid response time (<5 min), excellent selectivity against interfering biomolecules, and a detection limit in the low picomolar range (50 pg/mL), demonstrating potential for point-of-care endocrine diagnostics.

Didarian et al. [65] reported the selection of high-affinity DNA aptamers against PTH via SELEX and their application in an electrochemical impedimetric biosensor which is illustrated in Figure 3b. The aptamers were immobilized on a multi-wall carbon nanotube–modified gold electrode to form a sensitive interface for PTH binding. The recognition event altered the charge-transfer resistance, which was monitored using electrochemical impedance spectroscopy (EIS). The biosensor exhibited a linear response over 2–600 pg/mL with a detection limit of 0.42 pg/mL, showing strong selectivity and reproducibility in complex matrices. The device maintained over 90% of its initial response after 45 days of storage, highlighting its robustness for real-time PTH quantification and potential intraoperative or bedside use.

Xia et al. [66] designed a rapid intraoperative PTH assay based on the immune colloidal gold technique (ICGT) to facilitate real-time parathyroid identification during thyroid surgeries. The biosensor schematic and detection principle of that is presented in Figure 3c. The lateral-flow strip employs anti-PTH antibodies conjugated with colloidal gold nanoparticles to capture and visualize PTH in tissue eluates. The assay generates a visible test line within approximately 10 min, significantly reducing the turnaround time compared to conventional laboratory immunoassays. Clinical validation demonstrated high accuracy and concordance with standard PTH assays (sensitivity > 90%), supporting its application as a rapid, convenient, and low-cost diagnostic tool for intraoperative endocrine monitoring.

Collectively, these three studies illustrate the rapid evolution of parathyroid hormone (PTH) biosensing technologies toward greater sensitivity, speed, and clinical applicability. The innovations span diverse transduction strategies, from the dual optical-fluorescent AIE-LC polymer membranes enabling multi-channel, visual–quantitative detection, to aptamer-based electrochemical platforms offering exceptional sensitivity and stability, and immunochromatographic gold assays providing rapid intraoperative diagnostics. Together, they demonstrate a clear trend toward miniaturized, real-time, and point-of-care systems capable of supporting both laboratory and surgical environments. Future prospects include integrating these sensing mechanisms with microfluidic and wearable platforms, AI-driven signal analysis, and multiplex hormone detection to enable continuous endocrine monitoring. The convergence of optical, electrochemical, and immunological detection principles is expected to yield next-generation hybrid biosensors that combine high analytical performance with portability and user-friendliness, significantly advancing personalized diagnosis and intraoperative decision-making in endocrine disorders.

#### 2.1.4. *β*_2_-Microglobulin ($\beta_{2}M$)

Beyond electrolytes and hormones, *β*_2_-microglobulin, a protein usually filtered by healthy kidneys, accumulates in ESRD, forming amyloid fibrils that deposit in joints and soft tissues. This condition, known as dialysis-related amyloidosis, manifests as carpal tunnel syndrome, bone cysts, and spinal stenosis, severely impairing mobility and quality of life. Conventional dialysis membranes poorly remove *β*_2_-microglobulin due to its intermediate molecular weight, though high-flux dialyzers and hemodiafiltration have improved clearance. Despite these advances, *β*_2_-microglobulin remains a persistent toxin, highlighting the need for more efficient removal strategies to prevent long-term tissue damage [67,68].

The growing clinical importance of *β*_2_-microglobulin (*β*_2_M) as a biomarker in dialysis-related comorbidities, including amyloidosis and kidney dysfunction, has led to much innovation in the development of biosensing technologies which aim to support early diagnosis, real-time monitoring, and, in some cases, therapeutic interventions, providing a framework for managing chronic kidney disease (CKD) and dialysis-related complications.

**Figure 4:**
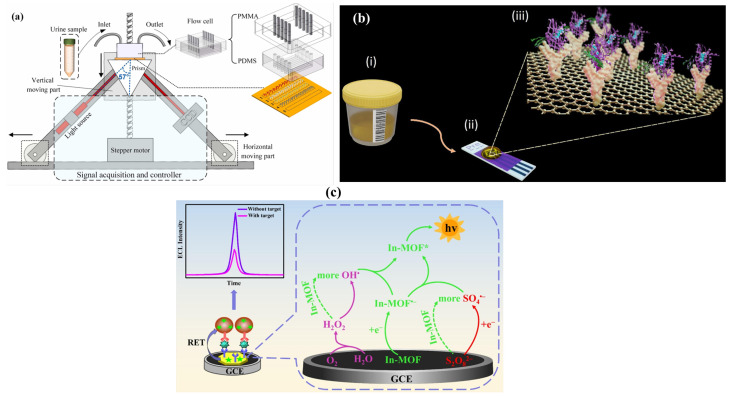
(**a**) Optical and angular scanning configuration of the SPRI instrument, illustrating the optical setup, mechanical framework, sensor chip, and the detection and reference channels [69]. (**b**) The 3-step protocol illustration of urinary β-_2_-microglobulin detection using an electrochemical immunosensor with a graphene working electrode functionalized with anti-β-_2_-microglobulin antibodies. (**i**) The urine sample is introduced onto (**ii**) an electrochemical screen-printed sensor, where (**iii**) the working electrode is modified with β-_2_-microglobulin-specific antibodies for target recognition [70]. (**c**) Schematic illustration of the electrochemiluminescence (ECL) detection mechanism based on In-MOF-modified glassy carbon electrode (GCE). The In-MOF facilitates redox reactions generating reactive species such as OH^·^ and ${\mathrm{SO}}_{4}^{\cdot -}$, enhancing ECL emission under light excitation (hν). The inset shows ECL intensity variation with and without the target, demonstrating the biosensor’s response [71].

One of the key label-free biosensing technologies employed in *β*_2_-Microglobulin detection is Surface Plasmon Resonance (SPR). Zhang et al. introduced a specialized multiplexed detection instrument for the simultaneous detection of four urinary proteins, including *β*_2_M [69]. This system (shown in Figure 4a) comprises a gold SPR chip functionalized using EDC/NHS chemistry and is divided into four microfluidic channels to allow simultaneous detection without cross-reactivity. The binding of *β*_2_M to their antibodies results in a change in the localized refractive index, which manifests as a change in the SPR wavelength, which in turn correlates with the analyte concentration. Using this method, this biosensor was able to detect *β*_2_M with a sensitivity down to 0.04 μg/mL, effectively distinguishing between healthy and nephrotic patients.

Electrochemical biosensors in recent times have provided low-power avenues for *β*_2_M detection. One such urine-based immunosensor proposed by Nasimi et al. [70] comprises a graphene screen-printed electrode functionalized with anti-*β*_2_M antibodies via a PBASE linker forming a sensing surface (demonstrated in Figure 4b). The binding of *β*_2_M creates an insulating layer, which results in a reduction of the electrochemical signal measured, which in turn is proportional to the *β*_2_M concentration. This method achieves an LOD of 204 μg/L and a total assay time of 45 min without sample pretreatment. Despite being created as a point-of-care diagnostic tool for patients with prostate cancer, this method’s strong applicability to *β*_2_M detection in CKD and dialysis highlights its potential in nephrology diagnostics.

Moreover, Hu et al. proposed an aggregation-induced emission electrochemiluminescence (AIECL) based metal–organic framework (IN-MOF) [71] which incorporates DPA ligands to overcome aggregation-caused quenching. The graphical abstract of the sensor is illustrated in Figure 4c. On applying a negative potential in the presence of co-reactant K_2_S_2_O_8_, the In-MOF generates strong luminescent emission. In the presence of *β*_2_M, a sandwich structure is formed between a capture antibody and a CeO_2_@PDA-labeled detection antibody. The latter reduces the ECL signal in proportion to the *β*_2_M concentration by acting as a quencher through resonance energy transfer (RET). This approach positions itself as a potential technique in the early diagnostics of renal disease by achieving a detection limit of 2.9 fg/mL with high specificity and dynamic range.

From optical and electrochemical biosensors to engineered peptide-based detection systems, recent biosensing techniques span various avenues. Their progression toward non-invasive, real-time, and accurate *β*_2_M detection indicates growing incorporation of biosensors into comprehensive dialysis patient management, both diagnostic and therapeutic.

#### 2.1.5. Creatinine

Creatinine, the most traditional biomarker of kidney function, remains a cornerstone of dialysis monitoring. As a byproduct of muscle metabolism, creatinine accumulates when renal clearance is impaired, and its levels are used to estimate dialysis adequacy (e.g., Kt/V, a measure of treatment effectiveness). However, creatinine has limitations: its production varies with muscle mass (leading to underestimation in malnourished or elderly patients), and it is poorly reflective of toxin buildup between sessions. Despite these drawbacks, creatinine remains indispensable for calculating urea reduction ratio (URR) and adjusting dialysis frequency, ensuring that treatments are sufficiently removing waste products [72,73,74,75,76,77].

Recent advancements in biosensor technologies for creatinine monitoring offer a unique approach to managing patients undergoing dialysis. As a key biomarker of renal function, creatinine levels provide critical insights into the efficacy of dialysis and the progression of kidney disease. However, conventional monitoring methods rely on intermittent blood sampling and centralized laboratory analysis, often delaying clinical response. New biosensor platforms are being developed to enable rapid, point-of-care, and continuous measurement of creatinine levels. These innovations allow for more timely and accurate assessment of renal clearance, supporting dynamic adjustments in dialysis therapy and enhancing individualized patient care.

One biosensor used to detect creatinine utilizes a laser diode-induced fluorescence detection system. A laser first excites fluorescent molecules, causing them to emit light at a higher wavelength. This emitted light is then captured and analyzed to determine both the presence and concentration of specific substances. When creatinine is mixed with 3.5-dinitrobenzoic acid (DNB), a fluorescent product is produced that absorbs UV light at 400 nm under highly alkaline conditions and emits fluorescence at 490 nm, which can be detected by using a fluorescence spectrophotometer to analyze the wavelength and determine the concentration of creatinine. Fluorescence detection of creatinine is determined by securing the excitation wavelength at 400 nm and monitoring the emission over time. A detection reagent is prepared by mixing LiOH (37.5 mmol/L, in distilled deionized water) with DNB (0.625 mol/L, in isopropyl alcohol) at 25 °C. A standard creatinine solution (0.3 mmol/L) is then introduced and shaken for 1 min before being transferred into a fluorescence detection setup. Fluorescence is recorded every second for 30 min. To evaluate the sensitivity, the process is repeated across a series of standard creatinine concentrations of 5 to 300 μmol/L. The resulting fluorescence intensities are plotted to create a calibration curve, which demonstrates strong linearity (R^2^ = 0.996) and a limit of detection (LOD) of 5 μmol/L [73].

Another biosensor that detects creatinine uses an electrochemical metallization (ECM) process. The sensing mechanism relies on the interaction between creatinine molecules and radical cations of the BOBzBT2 pentamer. When a forward voltage is applied, silver ions (Ag^+^) are generated at the working electrode and begin migrating toward the counter electrode. Simultaneously, BOBzBT2 radical cations are produced through charge transfer. Due to complementary electrostatic properties, these radical cations interact with creatinine molecules in the electrolyte. This interaction interferes with the transport of Ag^+^ ions, which disrupts the formation of the conductive silver filament between electrodes. When the conductive path does form, the cell shifts to a low resistance state, and when path dissolution occurs, it returns to a high resistance state. Increasing creatinine concentrations intensify this interference, reducing peak current and shifting the hysteresis behavior of the device. As a result, peak current shows a strong linear correlation with creatinine concentration. With an R^2^ value of 0.9597, the modified SPE sensor reliably measures creatinine concentrations between 0.7 and 1.1 mg/dL while maintaining good selectivity and reproducibility. Achieving an LOD of 0.36 mg/dL and an LOQ of 1.08 mg/dL, the sensor is validated for reliable detection within the necessary clinical range [76].

**Figure 5:**
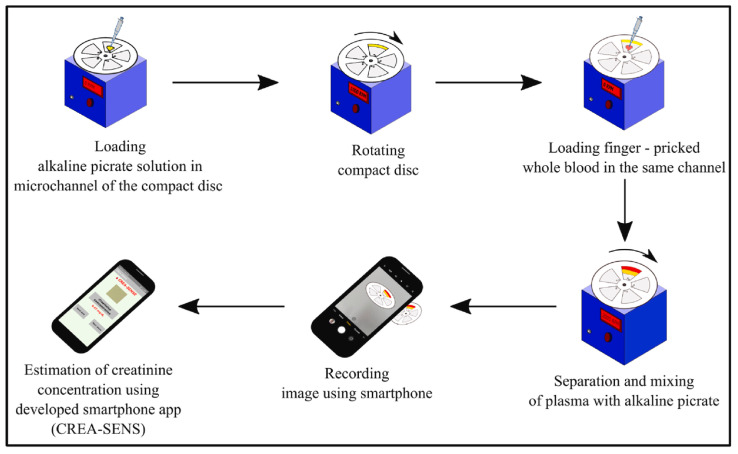
Step-by-step clinical assay of estimation of creatinine level on the developed device [77].

The last type of biosensor used to detect creatinine levels utilizes a lab-on-a-disc method for estimation of the concentration level of plasma-creatinine. As shown in Figure 5, the device, integrated with a spinning platform, operates using a DC motor controlled by a microcontroller, with a display showing the motor’s speed in rpm. Initially, 5 μL of alkaline picrate is loaded into a microchannel, and the disc is spun at 1000 rpm for 1–2 min to move the reagent toward the channel’s outer edge. Next, 10 μL of blood from a finger prick is added, and the disc spins at 1300 rpm for 10 min to mix the blood with alkaline picrate. This centrifugation step causes the separation of red blood cells from plasma, enabling a reaction between plasma creatinine and alkaline picrate, which forms a yellow-orange complex. The color change is captured using a smartphone camera inside a lightbox. A custom, in-house developed app called CREA-SENSE analyzes the image by performing region of interest (ROI) segmentation, background correction, and grayscale conversion. Image features are enhanced with spatial filtering, and the mean gray intensity at the detection zone is extracted. This intensity is compared to a pre-established calibration curve to determine creatinine concentration. The system is verified using 30 patient blood samples and analyzed through linear regression, which shows an agreement with standard clinical methods. The results yield a correlation of R^2^ = 0.998, which demonstrates the high accuracy and reliability of the device [77].

Incorporating real-time creatinine biosensors into dialysis treatment protocols has the potential to significantly improve clinical outcomes by providing immediate feedback on renal function. These systems can facilitate earlier detection of treatment inadequacies or emerging complications, allowing clinicians to intervene. As biosensor technology continues to mature, its integration into routine dialysis care may redefine monitoring standards, enabling more efficient, responsive, and personalized treatment strategies for patients with chronic kidney disease.

#### 2.1.6. Cystatin C

Cystatin C, another low-molecular-weight protein, has emerged as a more reliable marker of residual renal function and dialysis adequacy than creatinine in some cases. Cystatin C, unlike creatinine, is generated at a constant rate by every nucleated cell, unaffected by dietary factors or muscle composition, making it a more stable indicator of glomerular filtration rate (GFR). In dialysis patients, elevated cystatin C correlates with increased cardiovascular risk and mortality, possibly due to its association with inflammation and atherosclerosis. While not yet a routine test in all clinics, cystatin C offers a valuable tool for refining dialysis prescriptions, particularly in patients with atypical muscle mass or dietary habits that skew creatinine readings [78].

New biosensor technologies for monitoring cystatin C offer a promising advancement in dialysis care by enabling more precise and timely assessment of kidney function. Emerging biosensors are being developed to detect cystatin C rapidly and with high sensitivity at the point of care. These devices can provide feedback, allowing clinicians to adjust dialysis regimens more accurately and identify early signs of renal decline or recovery. By integrating cystatin C monitoring into dialysis protocols, these biosensors have the potential to personalize treatment schedules, reduce complications, and improve overall patient outcomes.

**Figure 6:**
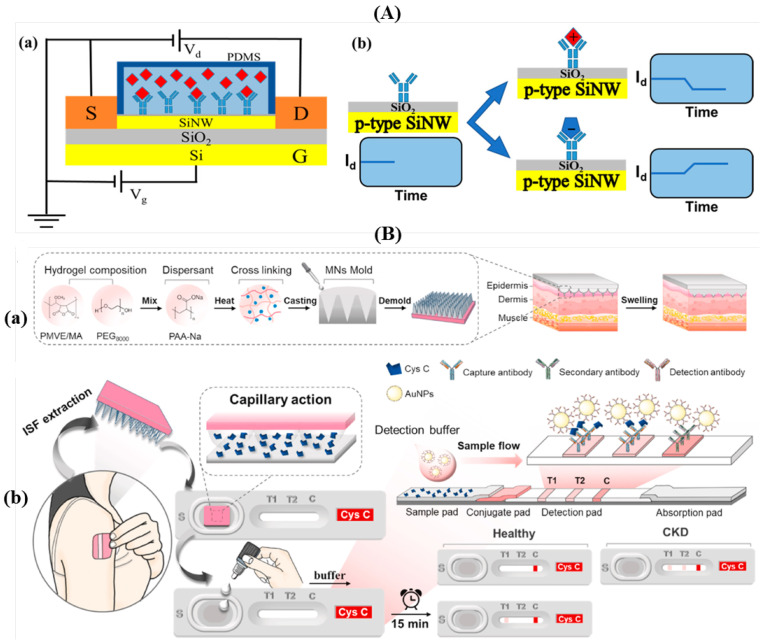
(**A**) Sensing mechanism of the SiNW FET biosensor. (**a**) Schematic representation of the structural configuration of the SiNW FET biosensor, and (**b**) Illustration of the detection principle of the p-type SiNW FET biosensor [80]. (**B**) Illustration of the fabrication and application of a microneedle (MN)-based hydrogel patch for noninvasive detection of cystatin C (Cys C). (**a**) Preparation process of hydrogel microneedle patches (HMNPs). (**b**) Schematic of the proposed blood-free rapid detection strategy for Cys C, showing interstitial fluid (ISF) extraction, capillary-driven sample flow, and detection modes for healthy and CKD subjects [81].

A new development of a monitoring technology for detecting urinary Cystatin C levels utilizes ultrasensitive silicon nanowires to determine early kidney failure. A Silicon Nanowire Field-Effect Transistor (SiNW FET) is a type of transistor that uses a silicon nanowire as the conducting channel. It operates the same as a MOSFET but with enhanced and unique properties due to its geometry and better electrostatic control. The nanowire surface is functional due to the receptors. When target molecules bind, they alter the surface charge, which modulates the channel conductance. The small changes can be picked up instantly, making it possible to detect biomolecules with great sensitivity [79]. The SiNW FET biosensor was fabricated in-house using an 8-inch CMOS platform at the Integrated Circuit Advanced Process (ICAC) Research and Development Center, Institute of Microelectronics, Chinese Academy of Sciences (IMECAS), Beijing, China. It detects Cystatin C (Cys-C) by measuring changes in current caused by molecular binding on its surface. As shown in Figure 6A, when positively charged Cys-C proteins attach to antibody-functionalized sites on a p-type SiNW, they reduce the hole carrier concentration, which then increases the resistance and decreases the current. This response intensifies with higher Cys-C concentrations, causing a leftward shift in the transistor’s transfer curve. In contrast, negatively charged molecules would increase hole carriers, boosting current. To assess the device’s sensitivity, varying concentrations of Cys-C (ranging from 1 ag/mL to 10 μg/mL) were applied, and both the $I_{d}-V_{g}$ characteristics and real-time current changes were monitored. The observed correlation between concentration and current change enabled quantification, with a calculated detection limit of 0.2529 ag/mL [80].

Another type of Cystatin C monitoring biosensor was designed using aptamers for rapid detection. This study introduces a lateral flow assay (LFA) for detecting Cystatin C (CysC) in human samples using an aptamer–antibody approach. An LFA is a diagnostic device used to detect the existence of a target analyte in a sample. The test starts with a sample like blood, saliva, or urine, which is applied to a sample pad and travels along the pad through capillary action. The pad contains antibodies attached to gold nanoparticles. If the target is existing, it binds to the labeled probes. The substance flows to the test line, where it comes across immobilized capture molecules that bind the target, forming a visible line if the target is present. The control line ensures the fluid flows properly and the test is valid. It should always appear, even if the test is negative [82]. To enhance sensitivity and specificity, CysC-targeting aptamers are labeled with the fluorescent dye AlexaFluor-647. (Molecular Probes™, Thermo Fisher Scientific, Carlsbad, CA, USA) In the presence of CysC, the aptamers bind the target and interact with immobilized antibodies at the LFA test zone, forming a fluorescent sandwich complex. Aptamers offer advantages such as ease of synthesis, scalability, and resistance to temperature changes because of their properties of being small and stable. Standard CysC solutions (0–120 μg/mL in PBS) are applied to the sensor and analyzed using the ImageQuant Immunoanalyzer. In samples without CysC, fluorescence only appears at the control line. When CysC is present, the test line also shows fluorescence due to aptamer–antibody binding. A clear, positive correlation between CysC concentration and signal intensity is observed, confirming accurate quantification. The assay demonstrates high precision and a detection limit of 0.013 μg/μL, exceeding traditional antibody-only kits [83].

The last type of biosensor that was developed utilized the lateral flow assay method again, using microneedle patches integrated with lateral flow cassettes for blood-free chronic kidney disease point-of-care testing. As demonstrated in Figure 6B, by merging a hydrogel microneedle patch (HMNP) and a lateral flow cassette (LFC), researchers developed a portable sensor for measuring Cystatin C (Cys C) in skin interstitial fluid (ISF). ISF, a fluid found between skin cells, contains biomarkers useful for noninvasive health monitoring. The sensor combines microneedle-based fluid extraction with a lateral flow immunoassay to allow rapid, at-home assessment of kidney function. The LFC includes a paper-based strip and a square sample port to receive the microneedle patch after skin application. To prepare the assay strip, the sample and conjugate pads are treated with PBS containing BSA and Tween-20, then dried. Goat anti-rabbit IgG is applied to create the control (C) line, while test lines T1 and T2 are formed using varying anti-Cys C antibody (AbCys C) concentrations, after which the entire detection area is dried. For testing, the HMNP is pressed onto the skin for 5 min to absorb ISF, then placed into the LFC sample port. ISF is transported through capillary action to the strip, after which a buffer solution containing HRP-conjugated gold nanoparticles attached to AbCys C is introduced. Over 15 min, the immunoreaction progresses, and results appear as red lines on the strip. In individuals with normal Cys C levels, the T1 line captures all complexes, showing only C or C/T1 lines. In those with elevated Cys C (as in CKD), excess complexes escape T1 capture, forming both T1 and T2 lines. This test produces visible results within 25 min and allows CKD patients to monitor their condition at home. Signal intensity at T1 increases with rising Cys C levels, and the T2 line appears only when concentrations exceed 4 μg/mL, indicating a clinically relevant threshold [81].

The integration of advanced biosensor technologies for cystatin C monitoring into dialysis care holds useful promise for enhancing clinical decision-making and patient management. By providing accurate, real-time insights into renal function, these systems can enable more personalized and responsive dialysis regimens. As biosensor platforms continue to evolve, their adoption in routine clinical practice may lead to earlier detection of renal deterioration, improved treatment efficacy, and ultimately, better patient outcomes in individuals with end-stage renal disease.

The buildup of these biomarkers, potassium, phosphorus, PTH, *β*_2_-microglobulin, cystatin C, and creatinine, paints a grim picture of the systemic havoc wreaked by kidney failure. Each compound contributes to distinct yet overlapping pathologies: potassium disrupts cardiac rhythms, phosphorus, and PTH destroy bones and blood vessels, *β*_2_-microglobulin cripples joints, and creatinine/cystatin C serves as surrogate measures of dialysis efficacy. Together, they underscore the importance of personalized, biomarker-guided dialysis regimens, where treatments are tailored not just to urea clearance but to the broader spectrum of uremic toxins. Future advancements in high-precision biosensors and high-flux filtration techniques may one day allow for real-time monitoring and targeted removal of these compounds, transforming dialysis from a blunt survival tool into a refined, life-extending therapy. Until then, vigilant biomarker management remains the cornerstone of reducing morbidity and mortality in ESRD.

### 2.2. Bacterial Biomarkers

Detecting infection early among dialysis patients is vital to increasing their odds of survival. Dialysis patients are 100 times more likely than regular individuals to develop staph bloodstream infections [84]. Recent research into bacteria detection with biosensors shows potential in protecting such a vulnerable group by implementing an electro-optical biosensor for passive detection [85], nanophotonic interferometric biosensors for rapid detection [86], and an electrochemical biosensor based on the facile synthesis of silver wire for detection across the membrane itself [87]. Infection detection in dialysis machines can be significantly optimized.

**Figure 7:**
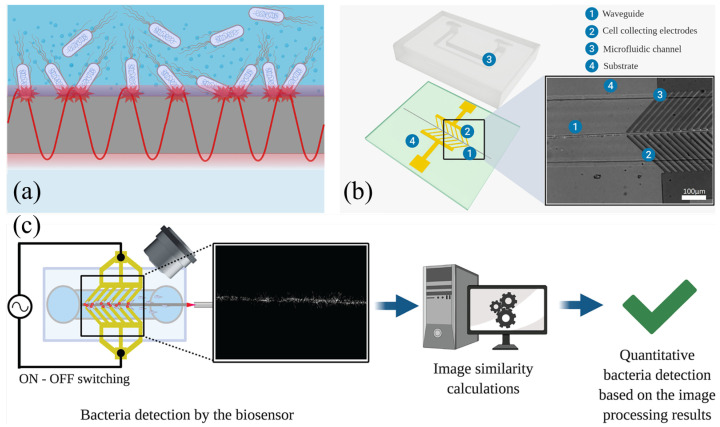
(**a**) The schematic illustration of the evanescent-field sensing, where the bonding of the *Escherichia coli* (*E. coli*) bacteria to the surface of the waveguide causes scattering of the evanescent waves of the light propagating in the waveguide. (**b**) The schematic design and realization of the integrated optical biosensor system. (**c**) Schematic demonstration of the high-level system diagram incorporating the evanescent-field sensor [85].

The first type of biosensor is an electro-optical biosensor that works by interpreting the light scattered by specific bacteria in the vicinity of a waveguide. Testing revealed a limit of detection of 10^2^ CFU/mL, which is enough to detect bacterial presence in body fluids. Dielectrophoretic collecting is used in tandem with this sensor to draw target analytes into the microfluidic channels. This magnetic force is calculated using the specific properties of the analyte that draw them toward the electric field maxima located in the channel. This property proves especially promising for utilization in dialysis, as it can be used to draw out bacteria in the blood into microfluidic channels located along the cellulose dialysis tubing. This would minimize further damage to the vital components of the blood by reducing interaction. Figure 7a demonstrates how *E. coli* bonding to the surface causes scattering of the evanescent waves in the waveguide (a channel where a controlled direction evanescent wave is located) [85].

As shown in Figure 7b, the biosensor itself is a thin-gold film electrode with tilted finger-like channels that optimize dielectrophoresis. The surface of the waveguide has electrodes created through soft lithography, which are treated with photopolymers and deionized to remove the exposed polymer. By covering the gold film with the specific surface charge, it is able to detect specific bacteria. Each channel leads to the waveguide strip located in the gap between the electrodes. During testing (Figure 7c), a laser diode is fed into the waveguide, and a function generator is attached to either end of the electrode clips of the sensor; analyte bonding signals cause scattering of the wave that is picked up by the CCD (Charge Coupled Device) camera attached to the microscope with greater visual stimuli indicating an increased presence of the target analyte (*E. coli*). This passive detection of bacteria would be critical in identifying infections at their onset, rather than waiting for more obvious symptoms to develop [85].

As opposed to the electro-optical sensor’s advantage in drawing in bacteria using dielectrophoresis, another biosensor is a nanophotonic interferometric sensor, which increases its specific response by rejecting bacteria. This is ideal for identifying specific types of bacteria, e.g., MRSA (Methicillin-resistant *Staphylococcus aureus*) and *Pseudomonas aeruginosa* (*P. aeruginosa*). It would be especially useful for determining the severity of infection and what treatment to pursue. Issues often arise in bacterial biosensors as bacteria naturally latch onto the sensor surface. The phosphate and carboxyl groups along their surface give them a slightly negative charge; this was utilized in the previous study to draw them in towards the electrodes. Having many different antigens present on one surface can lead to a drop in specific response. Treating the sensor surface with modified silane-PEG-COOH creates a slight negative charge that repels bacteria, preventing a rise in false positives. Maldonado et al. show that on the Si_3_N_4_ surface without treatment, many bacteria are present, as opposed to the case in which the surface is coated with silane-PEG-COOH, where significantly fewer bacteria are attached to the surface [86].

However, eventually, the target analyte is bound to the Silane-PEG-COOH and is treated with the given antibody and/or aptamer for detection. While the surface rejects bacteria, the antibodies covalently bonded to the carboxyl groups of the Silane-PEG-COOH draw them in. Similarly, aptamers were later employed by binding them to the amide group of the Silane-PEG-COOH. The detection of MRSA (Methicillin-resistant Staphylococcus Aureus) was extremely susceptible to being rejected without aptamer inclusion [86].

Moreover, similar to [85], a laser is run through the waveform; when it analyzes the bond to the target region, it causes scattering that is picked up by a photodetector. Detection of MRSA is essential for dialysis; it is most often contracted through invasive procedures such as surgery or intravenous tubing, and most cases of MRSA infection occur in nursing homes and dialysis centers [88]. It is especially deadly due to its antibiotic immunity and the current time needed for testing, with PCR taking anywhere from 6 to 7 h and cultures taking as many as 2 days [89]. Anophotonic interferometric biosensors provide a much faster solution, with results being detected in as few as 3–4 min. Employing this technology for specific and rapid detection could help individuals whose lives depend on the difference of a few hours.

While the electro-optical sensor can be placed along the intravenous tubing of a hemodialysis machine, and the nanophotonic interferometric biosensor can be placed at the initial injection site to test for specific bacteria such as MRSA, facile synthesis of silver wire across electrodes allows for detection across the dialysis membrane. Another group of biosensors relies on HCR (Hybridization Chain Reaction) in order to detect target bacteria, in this case, a small single-stranded DNA that is activated in the presence of an indicator (here, 16S rRNA, a common bacterial RNA component) and forms a long double-stranded DNA [90]. Two DNA hairpins (a single strand of DNA folded in on itself to form a “hairpin” structure), labeled H1 and H2-AuNPs (hairpin treated with gold particles), are placed on the electrode surface. Upon interacting with the target, they unfold and activate HCR, forming a double-stranded DNA. This process triggers the binding of silver nitrate injected onto the electrode to deposit onto the gold-treated DNA, creating a solid silver wire through deposition. This connects the two electrodes and causes a shift in the electrical parameters of the electrode that can be detected [87]. By placing either hairpin across the dialysis membrane, interaction with blood cells can be significantly reduced. This way, the wire only reaches across the membrane when bacteria are detected, as opposed to being ever-present across and causing further damage. Though it lacks a method of drawing bacteria in or detecting specific threats, being present across the membrane where all blood must pass through would enable passive detection without contributing significant damage.

Incorporating these biosensors into current dialysis technologies can help to significantly reduce mortality in an extremely vulnerable population. With infection being a major threat and individuals undergoing treatment being so vulnerable, it is important that detection is prioritized. In tandem, it is vital that membranes are minimally invasive when incorporating these sensors, as bio-incompatibility can lead to a rise in comorbidities that can further harm patients. The ideas put forward harness these values and allow for a safer device that can protect patients as well as aid them through treatment.

## 3. Bottlenecks of Current Technology

### 3.1. Potassium (K^+^)

Biosensor strategies for potassium monitoring demonstrate clear differences in sensing mechanisms, invasiveness, and long-term stability. The wrist-worn ECG–bioimpedance device developed by Rodrigues et al. [46] provides noninvasive, continuous interdialytic monitoring by correlating electrophysiological signal changes with serum potassium levels. Its main advantage lies in portability, patient comfort, and 72 h ambulatory tracking without blood sampling. However, because potassium is estimated indirectly through cardiac signal modulation, accuracy may be affected by patient-specific cardiac variability and comorbid conditions. In contrast, the anti-fouling iridium oxide microprobe developed by Wildner et al. [47] directly quantifies potassium electrochemically, achieving improved analytical precision and up to 28-day stability through zwitterionic anti-fouling coatings. While this approach enhances signal reliability and reduces drift compared to flexible printed circuit board (FPCB) systems, it introduces invasiveness and implantation-related risks.

The highly selective fluorescent potassium biosensor introduced by Depauw et al. [50] employs an optical sensing mechanism with strong ion selectivity, effectively minimizing sodium interference which is a common limitation in electrochemical potassium detection. This chemical specificity represents a significant improvement in molecular discrimination; however, optical systems require specialized instrumentation and careful calibration under physiological conditions. Collectively, these studies highlight a trade-off between noninvasive usability (wearable ECG systems), long-term electrochemical stability (iridium oxide microprobes), and superior ion selectivity (fluorescent probes). Although all approaches advance beyond traditional episodic blood testing by enabling continuous or real-time monitoring, challenges such as calibration drift, physiological interference, and long-term clinical validation remain key bottlenecks.

### 3.2. Phosphorus (Phosphate)

Phosphorus biosensing strategies demonstrate diverse transduction mechanisms with varying trade-offs in stability, detection limits, and clinical practicality. Zhang et al. inkjet-printed enzymatic sensor [58] offers a scalable and cost-effective platform capable of rapid detection (<10 s) with high sensitivity, making it attractive for point-of-care applications. Its printed architecture enables low-cost mass production; however, enzyme-based systems remain susceptible to activity loss, environmental instability, and biofouling in complex biological matrices. He et al. potentiometric copper ion-selective electrode (ISE) [59] eliminates the need for enzymatic reagents, providing faster response times and improved operational durability. While this approach enhances robustness and simplifies operation, it may experience interference from competing ions and matrix effects that impact long-term accuracy.

Cao et al. PhoR–PhoB whole-cell biosensor [60] introduces a biologically engineered sensing mechanism capable of nanomolar detection and exceptional specificity through intracellular regulatory pathways. This strategy achieves ultralow detection limits and strong selectivity, but it depends on maintaining cellular viability, which may limit long-term stability and clinical translation. Comparatively, the enzyme-based sensor provides manufacturing scalability, the ISE platform improves operational robustness and speed, and the whole-cell system offers superior sensitivity and specificity. Despite these advancements over conventional laboratory assays, challenges including biofouling, matrix interference, and stability under dialysis conditions remain key bottlenecks.

### 3.3. Parathyroid Hormone (PTH)

Recent PTH biosensors employ distinct molecular recognition and signal transduction strategies, each offering unique advantages over conventional ELISA-based laboratory assays. Tong et al. AIE-LC-Poly membrane platform [64] utilizes aggregation-induced emission (AIE) luminogens embedded within a liquid crystal–polymer matrix to generate amplified optical signals upon PTH binding. This approach enhances fluorescence sensitivity while enabling visual or optical readout with reduced background interference. Its main advantage lies in signal amplification and high sensitivity; however, optical systems may require controlled illumination conditions and careful calibration to maintain quantitative reliability in complex biological samples.

Didarian et al. aptamer-based impedimetric biosensor [65] replaces antibodies with synthetic aptamers, enabling label-free electrochemical detection through impedance changes at the electrode interface. This strategy improves chemical stability, reduces batch-to-batch variability, and allows miniaturization for point-of-care integration. Nevertheless, electrochemical platforms remain susceptible to surface fouling and signal drift over extended use. In contrast, Xia et al. ion-controlled gated transistor (ICGT) biosensor [66] leverages transistor-based signal amplification to achieve highly sensitive electronic readout, offering rapid response and strong signal-to-noise performance. While transistor-based systems provide superior electrical amplification and integration potential with wearable electronics, they require sophisticated fabrication and may face long-term stability challenges in biofluids. Collectively, these studies demonstrate improvements in sensitivity, miniaturization, and real-time detection compared to centralized immunoassays; however, stability, biofouling, and scalable clinical implementation remain persistent bottlenecks.

### 3.4. β_2_-Microglobulin (β_2_M)

Recent *β*_2_M biosensors employ optical, electrochemical, and luminescent amplification strategies to overcome the time constraints and centralized processing requirements of conventional immunoassays. Zhang et al. surface plasmon resonance (SPR) multiplex platform [69] enables label-free and real-time detection with picomolar sensitivity, allowing simultaneous monitoring of multiple analytes. The key advantage of SPR lies in its high specificity and kinetic binding analysis capability without the need for secondary labeling. However, SPR systems typically require bulky instrumentation and precise optical alignment, which may limit portability and point-of-care deployment.

Nasimi et al. graphene-based immunosensor [70] leverages the high surface area and exceptional electrical conductivity of graphene to achieve fg/mL level sensitivity through enhanced electron transfer at the electrode interface. This electrochemical approach offers miniaturization potential and rapid response (<10 min), making it attractive for dialysis monitoring. Nevertheless, graphene platforms may suffer from surface fouling and reproducibility challenges during large-scale fabrication. Hu et al. aggregation-induced electrochemiluminescence (AIECL) strategy integrated within a metal–organic framework (In-MOF) [71] further amplifies signal intensity, combining luminescent enhancement with structural porosity to improve detection sensitivity and selectivity. While this method provides ultrahigh sensitivity and strong signal amplification, it introduces material complexity and may require careful optimization for stability in biological fluids. These studies demonstrate significant improvements in detection limits and assay speed compared to standard laboratory immunoassays; however, instrumentation requirements, surface stability, and clinical integration into dialysis workflows remain ongoing bottlenecks.

### 3.5. Creatinine

Recent creatinine biosensors utilize optical, electrochemical, and centrifugal microfluidic strategies to overcome the time delays and laboratory dependence of conventional Jaffe or enzymatic assays. Chen et al. laser fluorescence-based sensor [73] employs fluorescence signal modulation for sensitive detection with μmol/L limits and strong linearity, enabling rapid and portable quantification. Its optical approach enhances sensitivity and reduces sample preparation requirements; however, fluorescence systems may require stable excitation sources and can be influenced by background autofluorescence in complex biological matrices.

Khushaini et al. electrochemical method (ECM) [76] provides label-free detection with fast response and good linear performance by leveraging direct electron transfer mechanisms. This strategy supports miniaturization and integration into portable devices but remains susceptible to electrode fouling and signal drift during prolonged exposure to serum samples. Ram et al. smartphone-integrated lab-on-a-disc platform [77] combines centrifugal microfluidics with optical readout, offering automated sample handling, portability, and user-friendly operation. While this system improves point-of-care adaptability and reduces operator dependence, it may involve greater mechanical complexity compared to single-electrode platforms. Collectively, these approaches demonstrate significant improvements in portability, response time, and real-time monitoring capability compared to centralized laboratory assays; nevertheless, long-term calibration stability, biofouling, and robustness in dialysis environments remain critical limitations.

### 3.6. Cystatin C

Emerging cystatin C biosensors employ transistor-based, lateral flow, and microneedle-assisted strategies to enable earlier and more sensitive assessment of glomerular filtration rate (GFR) compared to conventional laboratory immunoassays. Li et al. silicon nanowire field-effect transistor (SiNW-FET) platform [79] utilizes changes in surface charge upon antigen–antibody binding to produce highly sensitive electrical signals, achieving ultralow detection limits down to the ag/mL range. This label-free, real-time electronic readout offers rapid response and strong signal amplification, making it highly attractive for continuous or near-continuous monitoring. However, FET-based sensors can be sensitive to ionic strength variations and may require careful surface functionalization to maintain stability in complex biofluids.

Koczula et al. aptamer-based lateral flow assay (LFA) [82] provides a simple, low-cost, and user-friendly alternative suitable for point-of-care settings. Its advantages include rapid visual detection, portability, and minimal instrumentation requirements. Nonetheless, LFAs generally offer lower quantitative precision compared to transistor-based platforms and may face limitations in dynamic range. Chen et al. hollow microneedle patch (HMNP) approach [81] integrates minimally invasive interstitial fluid sampling with sensitive detection, enabling noninvasive or minimally invasive monitoring of cystatin C for GFR assessment. While this strategy enhances patient comfort and supports wearable integration, challenges such as long-term biocompatibility, biofouling, and calibration stability remain. Collectively, these platforms demonstrate substantial improvements in sensitivity and portability compared to centralized immunoassays, yet clinical robustness and long-term stability in dialysis populations continue to represent key bottlenecks.

A comparison of existing biosensing platforms for molecular and bacterial biomarkers is presented in Table 1, highlighting the sensing principles and key performance metrics such as detection limit, accuracy, and response time.

## 4. Future Prospects

Looking ahead, future prospects in biosensor development for dialysis management lie in integrating multi-analyte platforms that simultaneously monitor potassium, phosphorus, PTH, *β*_2_M, cystatin C, and creatinine, leveraging nanomaterials such as graphene or quantum dots to enhance sensitivity and antifouling performance. Advances in wearable and implantable technologies, combined with microfluidic integration into dialysis systems, could enable real-time feedback loops for automated treatment adjustments. Such systems may help mitigate dialysis-associated cardiovascular complications (hyperkalemia-induced arrhythmias and vascular calcification due to mineral imbalance) as well as bone metabolism disorders, thereby improving overall patient outcomes.

To address persistent bottlenecks in dialysis biosensing, particularly signal drift, biofouling, and calibration instability, emerging computational and materials-based strategies offer mechanistic solutions rather than merely incremental improvements. Artificial Intelligence (AI) and Machine Learning (ML) algorithms can be embedded into biosensing platforms to continuously analyze temporal signal fluctuations and distinguish true biomarker variation from baseline drift caused by temperature shifts, membrane degradation, or surface fouling. By training predictive models on historical calibration data, these systems can implement adaptive filtering, nonlinear regression correction, and real-time recalibration protocols, thereby compensating for progressive sensitivity loss during prolonged monitoring. Such closed-loop computational correction is especially relevant for wearable and implantable biosensors, where frequent manual recalibration is impractical.

In parallel, advances in nanomaterials and synthetic biology provide material-level strategies to enhance intrinsic sensor stability. Graphene and quantum dots exhibit high electron mobility and large surface-to-volume ratios, enabling improved electron transfer kinetics and enhanced signal-to-noise performance. Their tunable surface chemistry allows functionalization with antifouling coatings that reduce nonspecific protein adsorption, a primary contributor to baseline drift in complex biological fluids such as blood or dialysate. Furthermore, synthetic biology and CRISPR-engineered recognition elements offer programmable and highly selective binding architectures, minimizing cross-reactivity and signal interference from structurally similar analytes. By improving molecular specificity and reducing nonspecific interactions, these approaches contribute to long-term calibration stability and reproducibility.

Although the reported limits of detection (LoDs) of nanomaterial-based biosensors are often at the ultralow end, their clinical utility should be assessed relative to existing reference values. For example, in dialysis patients, the serum potassium concentration is known to vary from 3.5 to 6.5 mmol/L, while the decision limits of the levels of intact parathyroid hormone (PTH) are reported in the range of tens to hundreds of pg/mL, as suggested in the guidelines on CKD-MBD [91,92]. Similarly, the concentration of cystatin C is known to be above 1–5 mg/L in patients with advanced CKD [17,93], while *β*_2_-microglobulin can be raised above 20–30 mg/L in patients undergoing prolonged dialysis [94]. Thus, while conventional laboratory tests are used to measure these values, nanomaterial-based electrochemical and optical biosensors have been reported to have LoDs several orders of magnitude below these values, e.g., sub-pg/mL for PTH and fg/mL for cystatin C [79,82]. Thus, the role of nanomaterials is not only limited to improving the sensitivity of the biosensors but also extends to the overall performance in terms of an enhanced signal-to-noise ratio, dynamic range, and stability under continuous monitoring conditions. Together, computational intelligence and advanced materials integration represent complementary strategies to overcome drift-related limitations and enable reliable, real-time monitoring in dialysis management.

## 5. Conclusions

For patients with advanced kidney failure, dialysis is still a life-sustaining treatment, but it is unable to restore the intricate regulatory, endocrine, and immunological functions of the kidneys. Because of this ongoing biochemical and molecular dysregulation, dialysis patients have a high prevalence of comorbidities. In addition to being markers of renal dysfunction, this review emphasizes how biomarkers like potassium, phosphorus, parathyroid hormone, *β*_2_-microglobulin, creatinine, and cystatin C actively contribute to cardiovascular disease, bone pathology, inflammation, and long-term morbidity in this population.

Recent advances in biosensor technology show great promise in overcoming the limitations of traditional lab-based monitoring. Electrochemical, optical, nanophotonic, and biologically engineered biosensors now provide sensitivity, speed, and miniaturization, making real-time, point-of-care, and even continuous monitoring more achievable. Importantly, several platforms discussed here can work with wearable formats or be directly integrated into dialysis circuits. This development opens new opportunities for adjusting treatment dynamically during and between dialysis sessions. New infection-focused biosensors highlight the potential of these technologies to enhance early detection and prevention of life-threatening complications.

Despite this progress, significant challenges still exist before widespread clinical use can happen. Issues like long-term stability, biofouling, calibration drift, and compatibility with current dialysis systems must be carefully addressed. Additionally, turning high analytical performance into useful decision support will require linking with digital health platforms, using machine learning for data interpretation, and ensuring thorough clinical validation.

Looking ahead, the combination of biosensing, microfluidics, materials science, and precision medicine could change dialysis care from a reactive, intermittent model to a proactive and personalized approach. Multiplexed biosensors that can simultaneously monitor electrolytes, toxins, hormones, and inflammatory markers might allow for patient-specific dialysis prescriptions and early intervention strategies. As these technologies develop, biosensor-enabled monitoring could greatly lower complications, enhance quality of life, and set new standards of care for patients with chronic kidney disease and those who rely on dialysis.

## Figures and Tables

**Table 1:** Summary of biosensing approaches for detecting clinically relevant molecular and bacterial biomarkers associated with kidney function and infection monitoring.

Category	Biomarker	Biosensor	Working Principle	Limit of Detection	Accuracy	Test Time
Molecular	Potassium	Wrist/Patch-worn biosensor [46]	Optical Sensing (PPG Signal)	Not Reported	86%	Continuous monitoring during dialysis (∼3–4 h sessions)
		Iridium-oxide potassium-selective microprobe [47]	Electrochemical (Potentiometric)	0.5 mM	Not Reported	60 min
		Highly selective fluorescent potassium sensor [50]	Fluorescent	High selectivity for K^+^	Not Reported	Instantaneous (real-time monitoring)
	Phosphorus	Inkjet-printed pyruvate-oxidase reagentless enzyme-based biosensor [58]	Electrochemical	0.13 mM	98.9–103% recovery	Not Reported
		Copper nanoparticle-modified all-solid-state phosphate ion sensor [59]	Potentiometric and electrochemical	$1\times 10^{-6}$ M	10% RSD	<10 s
		PhoR–PhoB engineered *E. coli* whole-cell biosensor [60]	Electrochemical	0.012 mM	232-fold dynamic range	8 h
	PTH	AIE-LC-Poly liquid-crystal biosensor [64]	Optical and fluorescence sensing	0.01 μg/mL for optical detection and 50 pg/mL for fluorescence detection	96%	∼2 h
		Aptamer-based electrochemical impedimetric biosensor [65]	Label-free electrochemical impedance spectroscopy	20 pg/mL	98.75%	Instantaneous (real-time monitoring)
		Colloidal-gold labeled lateral-flow immunochromatographic assay [66]	Colorimetric	10 pg/mL	98.6%	2 min for tissue block homogenate (TBH) method (in vitro); 6 min for fine-needle aspiration (FNA) method (in situ).
	*β*_2_M	Portable multiplexed SPR biosensor [69]	Surface plasmon resonance	0.04 μg/mL	No difference with gold standard	4 min
		Anti-*β*_2_M antibody-functionalized graphene-based electrochemical immunosensor [70]	Label-free electrochemical immunosensing	204 μg/L	98.76%	45 min
		In-MOF AIECL electrochemiluminescence immunosensor [71]	ECL-RET immunoassay	2.9 fg/mL	101.9 ± 3.0%	∼2 h
	Creatinine	Fluorescence-based sensor (DNB reagent) [73]	Fluorescent	5 μmol/L	100 ± 15%,	∼30 min
		${\mathrm{BOBzBT}}_{2}$-Modified Screen-Printed Electrode (SPE) [76]	Electrochemical metallization (ECM)	0.36 mg/dL	95.97%	60 s
		Smartphone-Integrated Portable Rotating Platform [77]	Centrifugal colorimetry	0.1–15 mg/dL	99.8%	∼10 min
	Cystatin C	Ultrasensitive SiNW FET [80]	Electrical	0.25 ag/mL	98.7%	∼2 min
		Aptamer-Antibody Pair-Based Lateral Flow Assay (LFA) [83]	Fluorescent	0.013 μg/mL	99.26%	15 min
		Hydrogel Microneedle Patch (HMNP) and Lateral Flow Cassette (LFC) [81]	Sandwich fluorescence LFA	0.5 μg/mL	No difference with gold standard	25 min
Bacterial	*E. coli* DH5α	Integrated Electro-Optical Biosensor [85]	Dielectrophoresis-enhanced light scattering	$10^{2}$ CFU/mL	Not Reported	10 min
	*P. aeruginosa* & MRSA	Nanophotonic Interferometric Biosensor Based on Bimodal Waveguide Interferometer (BiMW) [86]	Bimodal waveguide interferometry	800 CFU/mL	98.46%	∼15 min
	16S Ribosomal RNA	Electrochemical Biosensor Based on Facile Synthesis of Silver Wire Across Electrodes [87]	Electrical	50 CFU/mL	95.63%	100 min

## Data Availability

No new data were created or analyzed in this study.
